# Home Working and Physical Activity during SARS-CoV-2 Pandemic: A Longitudinal Cohort Study

**DOI:** 10.3390/ijerph182413021

**Published:** 2021-12-10

**Authors:** Venerando Rapisarda, Carla Loreto, Laura De Angelis, Giuditta Simoncelli, Claudia Lombardo, Riccardo Resina, Nicola Mucci, Agata Matarazzo, Luigi Vimercati, Caterina Ledda

**Affiliations:** 1 Occupational Medicine, Department of Clinical and Experimental Medicine, University of Catania, Via Santa Sofia, 87 Building 10B, 95123 Catania, Italy; vrapisarda@unict.it (V.R.); riccardoresina@gmail.com (R.R.); 2 Human Anatomy and Histology, Department of Biomedical and Biotechnology Sciences, University of Catania, 95123 Catania, Italy; carla.loreto@unict.it; 3 Technological Innovation, Department of Technological Innovations and Safety of Plants, Product, and Anthropic Settlements, INAIL (National Institute for Insurance against Accidents at Work), 00144 Roma, Italy; la.deangelis@inail.it (L.D.A.); gi.simoncelli@inail.it (G.S.); 4 Human Anatomy, Department “G.F. Ingrassia”, University of Catania, 95123 Catania, Italy; claudialombardodoc@gmail.com; 5 Occupational Medicine, Department of Experimental and Clinical Medicine, University of Florence, 50139 Florence, Italy; nicola.mucci@unifi.it; 6 Department of Economics and Business, University of Catania, 95129 Catania, Italy; amatara@unict.it; 7 Occupational Medicine, Interdisciplinary Department of Medicine, Aldo Moro University of Bari, 70121 Bari, Italy; luigi.vimercati@uniba.it

**Keywords:** home work, remote working, sedentary time, fitness, workplace health promotion, COVID-19, pandemic

## Abstract

Background: Due to the SARS-CoV-2 pandemic, human lifestyles and occupational settings have changed in the workplace. This survey explores associations of home working employment and related physical activity (PA–MET min/week). Methods: A longitudinal cohort study was conducted between March 2020 and March 2021. A standardized method for assessing PA and sedentary time, the Italian version of the International Physical Activity Questionnaire-Short Form (IPAQ-SF), was used through the Microsoft Forms^®^ platform for self-administering the questionnaire. Baseline data were collected, and four follow-ups were performed; a full calendar year was observed. Results: In total, 310 home workers were recruited in this investigation. The average body mass index (BMI- kg/m^2^) was 21.4 ± 4.2 at baseline. The value increased at the first follow-up and fluctuated in the other recalls. The *t*-test of MET values of the four activities (Total PA, Vigorous-intensity activity, Moderate-intensity activity, Walking) show similar results; the total PA, at baseline 275.7 ± 138.6, decreased statistically significantly at the first (198.5 ± 84.6), third (174.9 ± 98.4), and fourth (188.7 ± 78.5) follow-ups, while it increased statistically significantly at the second follow-up (307.1 ± 106.1) compared to the baseline. Sedentary time was constant until the second follow-up, while it increased statistically significantly at the 3rd and 4th follow-up. Conclusion: workers involved reduced and reorganized their PA during this pandemic year. Each business company should intervene to improve the PA levels of workers and reduce sedentary behavior in the workplace.

## 1. Introduction

The SARS-CoV-2 pandemic has severely changed the working lifestyle. In order to reduce physical contact among workers and prevent new contagions, from 11 March 2020, enterprises urgently implemented “home working” or “remote working” due to the rising number of cases and regulations of the first lockdown (from 11 March to 4 May 2020) [1].

National policies forced social distancing, the closing of schools and universities, and the interruption of any community event. All activities practiced in gyms, sports centers, and swimming pools were banned. Furthermore, jogging or walking in parks and cycling were banned. Indispensable activities (farming, biomedical manufactory, information and communication technology, medical aid, energy production, and others) were continued, but, when possible, work (for example, administrative tasks and meetings) had to be performed from home, using internet connections and web conferencing tools. The priority was “stay at home” to reduce the spread of the virus [2].

Before then, Italy had the lowest number of remote workers throughout all of Europe (8%) [3], but through the SARS-CoV-2 pandemic, remote workers grew to 69% [4]. Several services were closed during the lockdown, and the community was advised to remain at home. In Italy, on 3 May 2020, outdoor places and green spaces reopened, applying only social distancing rules. Sports facilities reopened on 25 May 2020, but, due to the rising epidemiological conditions, were closed on 26 October 2020.

Physical activity (PA) is defined as the physical movement generated by skeletal muscles usage, causing an energy expenditure that is beyond the rest value [5], which involves some behaviors such as walking, dancing, or cycling [5,6,7,8,9]. Moreover, lockdown leads to physical inactivity, contributing to various health changes such as premature aging, obesity, cardiovascular vulnerability, muscle atrophy, bone loss, and decreased aerobic capacity [4,10,11]. It is well known that physical inactivity triggers over 5 million deaths worldwide and denotes damage to the economy of public health systems [2].

Moreover, restrictions may have reduced the motivation for daily PA [12]. Lack of sufficient PA is recognized as a severe public health issue that has been mainly questioned on several fronts [7]. Consequently, the World Health Organization (WHO) suggested 150 min of moderate-intensity aerobic PA every five days shared with at least two days per week of muscle strengthening to be judged as physically active and healthy [6]. These proposed standards are comparable to the individual’s health advantages, providing 10,000 steps per day [8].

The SARS-CoV-2 restrictions forced everyone to socially distance and self-isolate. Therefore, people spent more time at their residency, especially those working from home [10].

Records show consolidated studies about the effects of homeworking, triggering stress, anxiety, and isolation, which impacts on job effectiveness, well-being, and work–life balance [11,13,14]. However, to date, few studies have followed cohorts of workers who have approached work from home due to the COVID-19 pandemic. Regular PA is a key health behavior from a preventive point of view; for this reason, occupational physicians played a key role in the context of a comprehensive “system” responding to the pandemic. One of the traits is reorganizing working activities, ensuring workers’ safety and health are compulsory to avoid the onset of new epidemic outbreaks and provide a new preventive approach [15,16]. Evaluating how health risks and the benefits of home working are affected is key to best preserving occupational health. Workers or employers could not have anticipated the sudden shift to home working, so the safety of the home working environment has not necessarily been ensured [17,18].

This study aims to evaluate the fluctuations of PA in home working settings before, during, and after a year of the SARS-CoV-2 pandemic. Therefore, the present study has the objective of evaluating home working and giving some information on aspects related to health to understand the phenomenon and provide scientific evidence that may be useful for occupational physicians who deal with the health surveillance of home or remote workers.

## 2. Materials and Methods

### 2.1. Design and Selection of Study Subjects

A longitudinal cohort study was carried out to understand the variations in home workers’ lifestyles during the SARS-CoV-2 pandemic. This investigation was conducted between March 2020 and March 2021. The study was conducted as part of the periodic occupational health surveillance required by the Italian decree 81/08 [19], which provides routine medical examinations regularly, including specific medical screening tests. Occupational health surveillance can also take the method of periodic clinical and/or physiological evaluation of single workers. The benefit of this is detecting adverse health effects resulting from work-related exposures at an initial stage so that prompt, appropriate preventive measures can be instituted [20]. Regularly, these workers are visited upon recruitment (on this occasion, all personal data, mobile phone, and e-mail contacts are recorded) and, for the most part, subsequently reviewed every two years.

The workers of four private companies in Sicily (Italy) included in the survey were within administrative, secretarial, or organizational fields; therefore, they continued to execute working from home for the entire investigation period.

Inclusion criteria were: all office workers aged 30–55 years, working more than 36 h per week.

Exclusion criteria were: severe health conditions such as inflammatory disease or cancer, and reduced physical ability. Moreover, pregnant ladies were excluded. In addition, workers who contracted COVID-19 and those who were in quarantine were excluded from the study. Another exclusion factor was that the workers did not change the number of hours per week concerning the carried out work activity before the SARS-CoV-2 pandemic.

The employees that met inclusion criteria were contacted via e-mail by the occupational medicine unit of the University of Catania requesting an employee’s consensus. The first mail was sent on 14 March 2020. This represents the baseline data used in this investigation. A follow-up was conducted every 3 months via e-mail (14 June 2020, 14 September 2020, 14 December 2021, and 14 March 2021).

**Figure 1:**
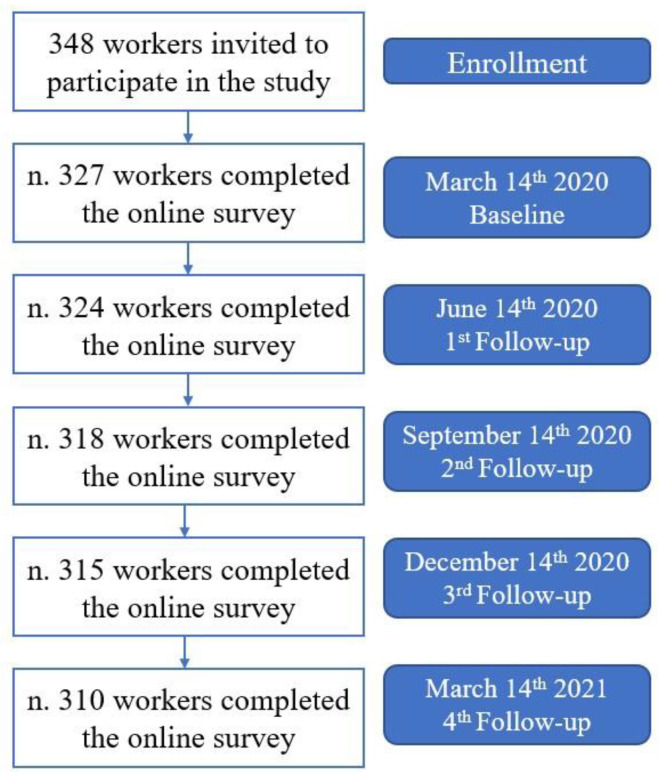
Baseline and follow-up flow chart.

The data relating to workers who did not continue the survey were not reported in the statistical analysis. The STROBE checklist was used for writing the present investigation (Figure 1) [21].

Workers were informed that data from the research protocol would be treated anonymously based on scientific methods and for scientific purposes following the principles of the Helsinki Declaration. Ethical approval is unnecessary because the study was performed according to Italian law concerning the protection of workers exposed to occupational risks [19].

### 2.2. Measures

All measures related to the worker’s entire day in the pandemic period, including working hours and activities performed on non-working days.

The Italian version of the International Physical Activity Questionnaire-Short Form (IPAQ-SF) [22,23], a standardized method for assessing PA, was used through a Microsoft Forms^®^ platform for self-administering the questionnaire. IPAQ-SF validity and reliability have been evaluated by Mannocci et al. [23].

Specifically, employees described the number of days and minutes/hours per day over the last 7 days that involved walking, moderate-intensity PA, and vigorous-intensity PA. Applying the IPAQ scoring procedures, Metabolic Equivalent for Task (MET) scores were calculated for walking, moderate-intensity PA, and vigorous-intensity PA using an automatic scoring tools in Microsoft Excel spreadsheet (License: CC BY-NC-SA 4.0) [24]. Based on the IPAQ-SF protocol [21], workers of the study were categorized in three different groups of PA considering the MET in/week of the sum of walking, moderate-intensity PA, and vigorous-intensity PA: low active (<600 MET min/week); moderately active (≥600 MET min/week) and high active (≥3000 MET min/week). Higher MET scores reveal more significant levels of PA in each domain. The MET scores for moderate-intensity PA and vigorous-intensity PA were used in the current study. Moreover, the IPAQ-SF made it possible to determine the sedentary lifestyle still using a tools spreadsheet [24].

At each follow-up, information on sociodemographic and occupational issues, body mass index (BMI), and working times (start and end of work; work duration; the number of breaks) were obtained. Data on BMI were partially self-reported. The invitation e-mail specified to measure their body weight after an overnight fast to minimize the risk of errors. Regarding their height, this was considered to be what was measured during the last surveillance visit with the occupational physician.

### 2.3. Statistical Analysis

Employee characteristics were examined using descriptive statistics with mean and standard deviation, with absolute and relative frequencies in the case of factor variables.

The normality assumption was examined by Shapiro–Wilk test. To test the mean discrepancy in total PA (MET min/week) and other PA activity investigated among baseline and four follow-up measures, a one-sided matching sample *t*-test was applied, and Bonferroni–Holm was performed to correct multiple comparisons. Repeated Measure ANOVA should be used to compare baseline and follow-ups.

All statistical analyses were performed by STATA/SE 16.0 (StataCorp, Burbank, CA, USA). The level of significance was set at *p* ≤ 0.05.

## 3. Results

In total, 348 (100%) workers met the requirements and received, via e-mail, a survey invite, and 327 (94%) joined the study. At the last follow-up, 310 (89%) workers were observed. Nine workers did not continue the survey, while the remaining eight were excluded because they contracted COVID-19 or because they were in fiduciary isolation (Figure 1).

The average age of the workers was 44.1 years at baseline, and 53% (n.164) were female. Most of the workers had a master’s degree 164 (53%), 41% (128) a bachelor’s degree, and only 18 (6%) a PhD.

The average BMI unit was 21.4 ± 4.2 at baseline; the value increased significantly from the first follow-up (*p* < 0.001) (23.8 ± 3.6; 22.1 ± 3.8; 22.5 ± 4.0; 22.9 ± 4.3, for 1st, 2nd, 3rd, and 4th follow-up, respectively). There was no statistical evidence for a difference in participants’ characteristics between baseline and follow-up.

The *t*-test results of the MET values of the four activities show similar adaptations for all PA activity (Table 1); the total PA decreased statistically significantly at the first, third, and fourth follow-ups, while it increased statistically significantly at the second follow-up compared to the baseline (*p* < 0.001).

There is a statistically significant decreasing difference between the baseline and the first and third follow-up in the MET of vigorous-intensity activity (*p* < 0.001). In contrast, at the second follow-up the activity increases, while the MET of Moderate-intensity activity increases with a statistically significant difference between the baseline and the first and third follow-up. The statistically significant difficulty is negative between the baseline and the first, third, and fourth follow-up regarding the walking activity. In contrast, it is positive between the baseline and the second follow-up.

Sedentary life was constant until the second follow-up, while it increased statistically significantly in the 3rd and 4th follow-up. Table 1 shows in detail all the characteristics of the results.

## 4. Discussion

Since the COVID-19 pandemic, home working has become common for many office-based workers [25], and for those involved in this survey, it has probably been the first working from home experience.

This study highlights that self-reported PA decreased significantly during the lockdown, with a statistically significant increase in the summer (2nd follow-up) when sports centers reopened. After this period, there was a statistically significant decline until the last follow-up. This may have increased BMI, which improved during the first follow-up but remained constant throughout the pandemic year. The trend of walking activity was identical to that of total PA. Instead, vigorous- and moderate-intensity activity had a fluctuating trend, often in line with the baseline value. Regarding the daily sitting time, a statistically significant difference was instead found starting from the 3rd follow-up.

To our knowledge, there are no studies that have carried out a one-year working from home survey. However, a limited number of previous investigations have evaluated the impact of confinement caused by the SARS-CoV-2 pandemic on self-reported PA in workers but for a limited period. In addition to confirming what was reported by other investigations [24,25,26,27], such as the reduction in PA in the pandemic period, our study demonstrates the temporal modulation linked to the waves of COVID-19 disease in home workers.

A systematic review carried out by Zaccagni et al. [2] on PA during the two-month lockdown evidenced a harmful effect on general health in Italians, especially those with chronic conditions such as obesity and neurological diseases. Generally, a reduction in PA was identified as a concern of COVID-19 lockdown, with a deterioration in health status [2].

Significant reductions in PA due to the COVID-19 pandemic have been described, and it is expected that the pandemic situation may have a comparable impact on PA across employment groups [26]. Moreover, due to the increased sedentary time, it is reasonable that the positive fluctuations we have detected in PA are a possible fear as PA has been proven to benefit cardiometabolic health and may decrease overall mortality risk [27].

An investigation carried out among home workers based in Sweden during COVID-19 outbreaks, using an accelerometer to assess physical behavior for seven consecutive days, did not show an increase in sedentary time but did detect increased sleeping time [25]. This result differs from our study, probably due to the temporal diversity of the association and the fact that the Swedish government has not applied restrictive policies as in Italy. On the other hand, another investigation in Northern Italy during the SARS-CoV-2 pandemic registered that new home workers had further self-reported sedentariness compared with working in the pre-pandemic era, and they had drastically reduced their PA (68%) [28]. Nevertheless, among our workers, PA behaviors might have altered due to the pandemic. Workers who switched to home working due to the COVID-19 pandemic highlighted a more significant amount of time spent in sedentary behavior [28].

The results of our study reported an increase in sedentary time among home workers; it is well known that an increase in sedentary behaviors is among the most important public health factors due to their unfavorable physical and mental health impacts [29,30,31,32]. Employers should preserve employees’ health, thus increasing work productivity and preventing sick days [33].

Consequently, our results recommend that employer’s policies promote PA to workers operating remotely or in the office. In detail, these activities should be combined with psychological and motivational support since Zaccagni et al. [2] highlighted a connection between the psychology, motivation, and PA. Higher anxiety scores during the COVID-19 lockdown negatively motivated people to take part in PA [2].

Regarding moderate-intensity activity, our results indicate that it increases with consistency; this could be ascribed to the fact that workers may have been encouraged about the need to keep a minimum PA by social media and other interconnected virtual environments. It might also be noted that the less active workers could increase their moderate activities during home working, which could be associated with the sponsorship of such behaviors by health promotion institutions, fitness centers, the web, and TV by publishing routine online workout session schedules.

In this regard, promotional activities should be accompanied by accurate information about the risks related to exercise and health conditions and the specific worker’s needs, where moderate physical activity is conducted independently. Moreover, motivational action for PA behavior could support the change in leisure time PA intentions [34].

The strength of this study is having a survey that lasts a whole year and from the beginning of the pandemic. A limit of the current investigation is that the IPAQ-SF is a replacement for the IPAQ-Long survey due to fears that the questionnaire’s duration would cause a significant reduction in the number of participants. In addition, some assessments were made independently by the workers, such as weight. Finally, the effects of other lifestyles have not been evaluated.

PA is a precondition for health promotion, prevention, and therapy of several medical disorders, such as metabolic, cardiovascular, and neurodegenerative disorders.

Home working maintained in the long period may result in a risk factor of the deterioration of general health.

## 5. Conclusions

In conclusion, workers involved reduced and reorganized their PA during this pandemic year but require mandatory employer interventions to improve their PA levels and reduce sedentary behaviors within the workplace. Remote work will undoubtedly be present even more after the pandemic period. Therefore, occupational medicine should also pay attention to health promotion activities for those who work from home.

## Figures and Tables

**Table 1:** Sociodemographic characteristics of workers participating in the survey and IPAQ-SF results.

	Baseline 14 March 2020	1st Follow-Up 14 June 2020	2nd Follow-Up 14 September 2020	3rd Follow-Up 14 December 2020	4th Follow-Up 14 March 2021
Age (Mean ± SD)	44.1 ± 8.3				
Gender (Female-%)	164 (53%)				
Education level					
*Bachelor’s Degree*	128 (41%)				
*Master’s Degree*	164 (53%)				
*PhD*	18 (6%)				
Working age	13.9 ± 6.7				
BMI (Mean ± SD)	21.4 ± 4.2	23.8 ± 3.6 °	22.1 ± 3.8 °	22.5 ± 4.0 °	22.9 ± 4.3 °
Total PA * (Mean ± SD)	275.7 ± 138.6	198.5 ± 84.6 °	307.1 ± 106.1 °	174.9 ± 98.4 °	188.7 ± 78.5 °
Vigorous-intensity activity * (Mean ± SD)	69.4 ± 27.9	51.1 ± 33.4 °	74.5 ± 31.9	50.4 ± 22.5 °	60.3 ± 24.9
Moderate-intensity activity * (Mean ± SD)	129.7 ± 64.2	148.5 ± 84.6 °	144.2 ± 73.4 °	137.2 ± 44.6	134.8 ± 38.9
Walking * (Mean ± SD)	214.2 ± 89.0	165.8 ± 101.8 °	243.1 ± 112.8 °	137.9 ± 97.4 °	148.0 ± 104.5 °
Daily sitting time in hours (Mean ± SD)	8.4 ± 2.1	8.2 ± 4.4	8.6 ± 4.0	13.4 ± 5.3 °	14.0 ± 4.8 °

* MET minutes/week. ° difference statistically significant (*p* < 0.05; *p* < 0.001) compared to baseline.

## Data Availability

The data presented in this study are available on request from the corresponding author.
